# Internet use and frailty in middle-aged and older adults: findings from developed and developing countries

**DOI:** 10.1186/s12992-024-01056-6

**Published:** 2024-07-03

**Authors:** Liang Li

**Affiliations:** Department of Health Statistics, School of Public Health, China Medical University, ShenYang, China

**Keywords:** Internet use, Frailty index, Middle-aged and older adults, Multicohort, Mediate analysis

## Abstract

**Background:**

With increasing trend of internet use in all age groups, whether internet use can prevent frailty in middle-aged and older adults remains unclear.

**Methods:**

Five cohorts, including Health and Retirement Study (HRS), China Health and Retirement Longitudinal Study (CHARLS), the Survey of Health, Ageing and Retirement in Europe (SHARE), English Longitudinal Study of Aging (ELSA), and Mexican Health and Aging Study (MHAS), were used in this study. Internet use, social isolation, and frailty status was assessed using similar questions. The Generalized estimating equations models, random effects meta-analysis, COX regression, and mediation analysis were utilized.

**Results:**

In the multicohort study, a total of 155,695 participants were included in main analysis. The proportion of internet use was varied across countries, ranging from 5.56% in China (CHARLS) to 83.46% in Denmark (SHARE). According to the generalized estimating equations models and meta-analysis, internet use was inversely associated with frailty, with the pooled ORs (95%CIs) of 0.72 (0.67,0.79). The COX regression also showed that participants with internet use had a lower risk of frailty incidence. Additionally, the association was partially mediated by social isolation and slightly pronounced in participants aged 65 and over, male, not working for payment, not married or partnered, not smoking, drinking, and not co-residence with children.

**Conclusions:**

Our findings highlight the important role of internet use in preventing frailty and recommend more engagements in social communication and activities to avoid social isolation among middle-aged and older adults.

**Supplementary Information:**

The online version contains supplementary material available at 10.1186/s12992-024-01056-6.

## Introduction

Frailty, characterized by a decline in physical function across multiple physiology systems, is a worldwide burden with main implications for clinical practice and public health. With the progress of population aging, related financial costs and uses of medical resources have to be further aggravated because all older adults are vulnerable to developing frailty [1]. People with frailty are more like to increase the risk of adverse outcomes, including diabetes, cardiovascular disease, and mortality [2, 3]. Although diverse evaluation criteria about frailty status were reported [4–6], a frailty index, using a series of age-related health deficits, had an amply robust performance in predicting frailty status compared to other instruments [7]. Additionally, recent studies found that frailty was a dynamic process over time and could be transformed into non-frailty by specific interventions and prevention actions [1, 8]. Therefore, identifying the potential factors to prevent the incidence of frailty is urgent and crucial, which would protect the target population from suffering.

Over the past decade, the increasing trend of internet use was unstoppable in all age and gender groups while middle-aged and older adults remained the largest proportion of the digital divide [9, 10]. Digital technology is in favor of employability and communication for the individual of digital inclusion but provides few benefits to those with digital exclusion [11]. A growing number of studies reported that internet use was associated with the incidence of chronic diseases, healthy status, functional limitations, cognition declines, and depression [9, 12–15]. Those health deficits could be used to construct a frailty index according to a standard procedure, [4] but few studies have focused on the health effect of internet use on frailty. A prior study conducted a similar analysis to explore the protective effects of internet use on frailty based on frailty phenotype assessed by weight loss, exhaustion, weakness, slow walk speed, and low physical activity [16]. However, the cross-sectional design and small sample size limited the generalizability of the final findings. Besides, the pattern of association of internet use and frailty might be different between developed countries and developing countries as the rate of internet coverage varied tremendously across the countries [13]. Furthermore, possible mechanisms by which internet use reduced the incidence of frailty were unclear although some reasonable explanations were that the internet could provide healthcare services, maintain social connection, and decrease loneliness [17–20]. Previous studies indicated that internet use could improve health status and reduce depression mediated by social participation [21, 22]. Meanwhile, people with internet use were more likely to participate in social activities including participation in social clubs, religious activities, voluntary groups, life-long learning, and exercise clubs [23]. , and then engagement in social activities was associated with a lower risk of frailty [24]. So it was necessary to figure out whether social isolation, the opposite of social engagement, mediated the association between internet use and frailty status.

To fill the knowledge gaps, we performed a cross-cultural and muti-cohorts longitudinal analysis to explore the association of internet use and frailty status among middle-aged and older adults from global perspectives, as well as the mediating role of social isolation, based on large sample sizes from developing countries including China and Mexico and developed countries including the United States, England, Israel, and 27 European countries.

## Methods

### Study design and population

Data were obtained from five international longitudinal cohorts targeting middle-aged and older populations: Health and Retirement Study (HRS), China Health and Retirement Longitudinal Study (CHARLS), Survey of Health, Ageing and Retirement in Europe (SHARE), English Longitudinal Study of Ageing (ELSA), and Mexican Health and Aging Study (MHAS) [25–29]. These surveys were intended to provide comparable results for addressing aging issues, which all provided information on internet use, social isolation, and frailty index. In this study, data from approximate time ranges were used, namely, wave 11-wave 14 (2012–2018) for HRS, wave 1-wave 4 (2011–2018) for CHARLS, wave 5-wave 8 (2013–2019) for SHARE, wave 6-wave 9 (2012–2018) for ELSA, and wave 3-wave 5 (2012–2018) for MHAS.

We only included participants aged 50 and over, and further excluded those with missing information regarding internet use, frailty index, or covariates. Hence, 62,932 observations (23,074 participants) from HRS, 36,866 observations (17,690 participants) from CHARLS, 124,926 observations (87,517 participants) from SHARE, 27,146 observations (9869 participants) from ELSA, and 34,273 observations (17,545 participants) from MHAS were available for the main analysis (Supplementary Fig. 1). To explore the mediating effects of social isolation, participants with missing information on social isolation were also further excluded.

Since we used secondary-analysis data from public datasets, including HRS (approved by National Institute on Aging and the Social Security Administration, NIA U01AG009740), CHARLS (approved by the Ethical Review Committee of Perking University, IRB00001052-11015), SHARE (approved by the Ethics Council of Max Planck Society), ELSA (approved by National Research and Ethics Service Committee South Central-Berkshire), and MHAS (approved by National Institutes of Health/National Institute on Ageing, NIH R01AG018016), So the detailed ethical approval could be found on respective origin surveys. Meanwhile, written informed consent was also obtained from any participant.

### Measures

#### Exposure variable

Information on internet use was collected through self-reported questionnaires. In HRS, participants were asked: ‘‘Do you regularly use the Internet (or the World Wide Web) for sending and receiving e-mail or for any other purpose, such as making purchases, searching for information, or making travel reservations?”. In CHARLS, participants were asked whether they use the internet in the past month. In SHARE, the independent variable was constructed based on the following question: “In the last 7 days, have you used the Internet at least once for e-mailing, searching for information, making purchases, or for any other purpose?”. In ELSA, the participants were asked about the frequency they use the internet, and the responses ranged from 1 = ‘‘Every day, or almost every day’’ to 6 = ‘‘Never.’’ In MHAS, participants were asked whether they could assess internet service at home. The response “yes” (HRS, CHARLS, SHARE, and MHAS) or a frequency of at least once a week (ELSA) was classified as internet use, while the response “no” or a frequency of less than once a week was defined as digital exclusion [10]. 

#### Outcome variable

Frailty was evaluated by the frailty index, which was calculated as the accumulation of age-related health deficits [4]. According to previous research, we chose some items that could be available for five cohorts [24, 30]. After screening the data, 30 items in HRS, CHARLS, SHARE, and ELSA, and 28 items in MHAS were selected to construct the frailty index, which included multiple chronic diseases, self-reported healthy status, functional limitations, depression, and cognition (Supplemental Table 1). Participants with any missing items of 30 items in HRS, CHARLS, SHARE, and ELSA, and 28 items in MHAS were excluded from the procedure of computing the frailty index.

Most items were categorized as 0 or 1 according to the specific cut-off value. The value of 0 indicated the absence of the deficit, and 1 indicated the presence. Some conditions including self-reported general eyesight, hearing, healthy status, and cognition were in the range of 0 to 1, the high value indicated a worse deficit. In our study, the frailty index was calculated as the sum of present deficits divided by 30 or 28 and multiplied by 100. Therefore, the frailty index was theoretically a continuous variable ranging from 0 to 100. In our study, frailty status was a dichotomous variable including frailty and non-frailty. According to the suggestion of previous research [3, 30], frailty was defined as the frailty index ≥ 25, while non-frailty was the frailty index < 25.

#### Mediation variable

We created an index of social isolation using a 5-item scale according to previous studies (Supplementary Table 2) [31–33]. Participants were assigned a social isolation score based on these criteria: (i) unmarried, (ii) lived alone, (iii) less than weekly contact with children, (iv) less than weekly contact with parents, relatives or friends, (v) did not participate in any groups, clubs, or other organizations in the past month (HRS, CHARLS, and MHAS), past year (SHARE and ELSA). We only calculated scores for participants providing information for at least 3 of 5 items. Hence, the score range was 0 to 5, with higher scores meaning greater social isolation. We followed the previous study [32] to define participants in the top quintile of the score as social isolation (≥ 2 in HRS, CHARLS, ELSA, and MHAS, ≥ 3 in SHARE).

#### Covariates

Covariates were identified through literature reviews. We included age, gender, educational levels, work for payment, married or partnered, household wealth, smoking, drinking, and co-residence with children. Age was calculated by the time of the interview and birth date. The gender was reported as male or female. According to the International Standard Classification of Education 1997, educational levels were a three-tier variable, including less than high school, high school, and university and above. Work status was identified as whether the participants engaged in work for payment. Marital status was a binary variable of whether or not participants were married or partnered. The level of household wealth has been divided into tertile levels of non-housing financial wealth. Smoking was described as current smoking behavior while drinking was about whether participants ever consumed any alcohol. Co-residence with children was viewed as a binary question, and children missing was viewed as no co-residence.

### Statistical analysis

All continuous variables were described as mean ± standard deviation or median (Q1-Q3), and categorical variables were reported as numbers (percentages).

To handle the correlation across repeated measures, we utilized generalized estimating equations (GEE) models fitting with logit distribution with unstructured correlation and calculated odd ratios (ORs) and 95% confidence intervals (CIs) to investigate the association of internet use with frailty status in middle-aged and older population. We used three models in the analysis, of which model 1 was a crude model. In model 2, we accounted for age and gender. Model 3 was fully adjusted, further controlling educational levels, work for payment, married or partnered, household wealth, smoking, drinking, and co-residence with children.

To ensure the robustness of our findings, we did six sensitivity analyses. Firstly, we repeated the GEE models with the continuous frailty index as the outcome variable. Secondly, we exclude separately the participants with severe cognitive impairment and memory disease to reduce the recall bias. Thirdly, we conducted inverse probability weights GEE models to deal with biases due to dropout. Fourthly, E-values for exposure-outcome association were calculated to quantify unmeasured confounding based on the risk ratio scale. Fifthly, we further explore the association of internet use and specific domains, including general health, physical function, depression, and cognition, of frailty. Finally, to avoid possible reverse causation, we used a COX regression to further discover the association between internet use and frailty status after excluding participants with frailty at baseline.

We performed stratified and multiplicative interaction analyses by some covariates as modifiers to assess the potential difference in the effect of internet use across different subgroups. The GEE models were repeated after stratifying by age, gender, work for payment, married or partnered, smoking, drinking, and co-residence with children, respectively. The product terms of internet use and those covariates (e.g., internet use × age) were constructed to assess the multiplicative interaction in Model 3.

Furthermore, the mediating effect of social isolation in the association of internet use with frailty status was explored using the mediation analysis. The mediation proportion was calculated by dividing the indirect effect by the total effect. Directed acyclic graphs were used to visualize the results.

Random-effects meta-analysis was used to pool the ORs and corresponding 95% CIs from five cohorts to derive overall effect estimates. The heterogeneity test of effect estimated across cohorts was conducted using Cochran Q test.

All the analyses were performed in SAS 9.4 and Stata 18. The statistical significance of the study was determined with a two-sided P-value < 0.05.

## Results

**Fig. 1:**
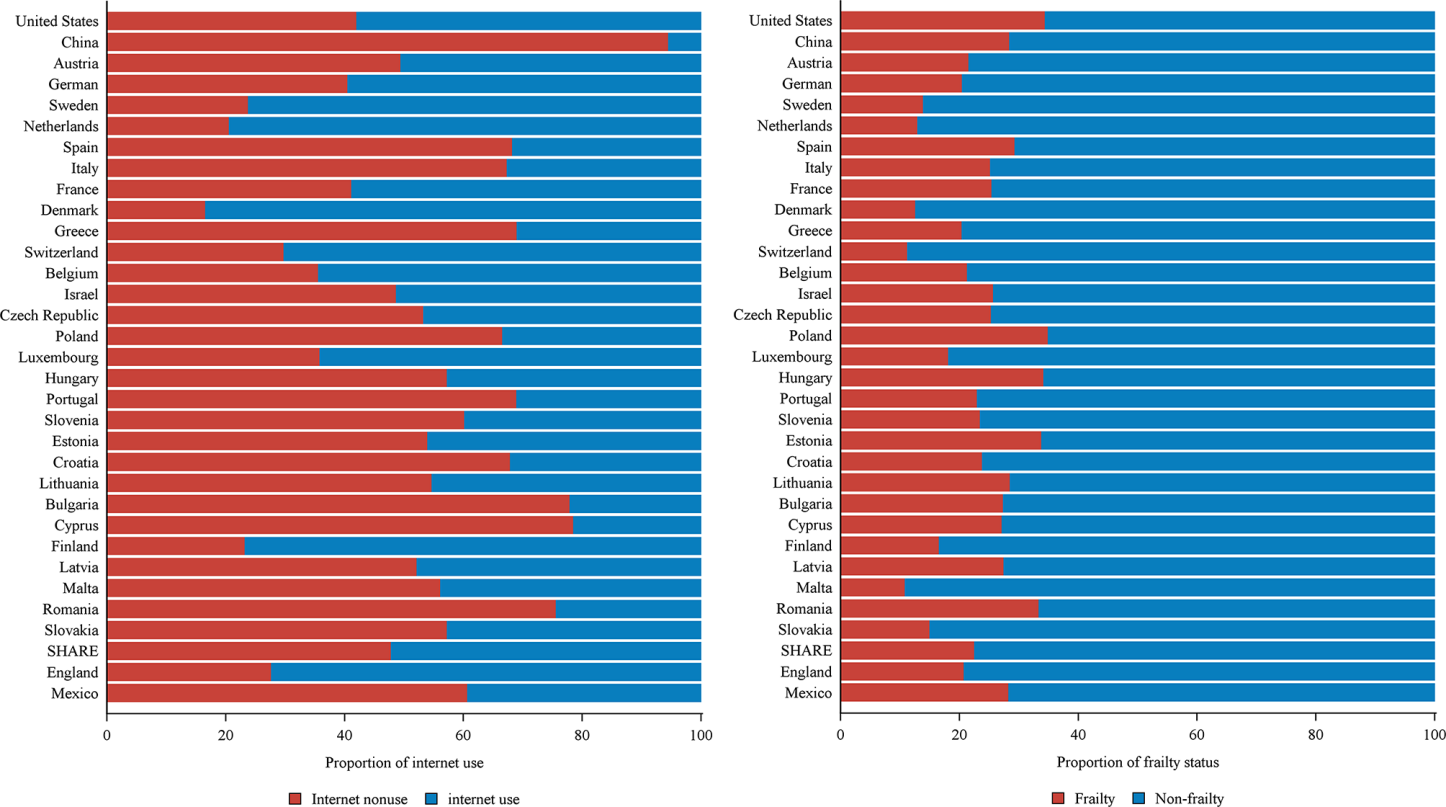
The proportion of internet use and frailty status

Table 1 shows the characteristics of observations in the five cohorts. The mean ages of HRS, CHARLS, SHARE, ELSA, and MHAS ranged from 62 to 68 and the male participants were about 43%. The median scores of frailty index were in the range of 12 to 18. (Supplemental Fig. 2) More than 65% of the participants reported non-frailty according to repeated measurements. The proportion of internet use varied widely across countries, ranging from 5.56% in China (CHARLS) to 83.46% in Denmark (SHARE). Additionally, the overall proportion of internet use in SHARE was 52.21%, while the rate was 58.01% in HRS, 72.35% in ELSA, and 39.37% in MHAS (Supplemental Tables 3 and Fig. 1).

**Table 1:** Descriptive statistics in HRS, CHARLS, SHARE, ELSA, and MHAS

	HRS (*N* = 62,932)	CHARLS (*N* = 36,866)	SHARE (*N* = 124,926)	ELSA (*N* = 27,146)	MHAS (*N* = 34,273)
Age	66.24 ± 10.28	62.32 ± 8.37	68.00 ± 9.75	67.65 ± 9.25	65.15 ± 9.42
**Gender**					
Male	25,928(41.20)	17,418(47.25)	54,307(43.47)	12,325(45.40)	14,404(42.03)
Female	37,004(58.80)	19,448(52.75)	70,619(56.53)	14,821(54.60)	19,869(57.97)
**Educational levels**					
Less than high school	10,188(16.19)	32,786(88.93)	48,207(38.59)	6677(24.60)	28,786(83.99)
High school	37,270(59.22)	3554(9.64)	48,189(38.57)	14,546(53.58)	1209(3.53)
University and above	15,474(24.59)	526(1.43)	28,530(22.84)	5923(21.82)	4278(12.48)
**Work for payment**					
No	36,663(58.26)	13,613(36.93)	86,417(69.17)	18,435(67.91)	20,782(60.64)
Yes	26,269(41.74)	23,253(63.07)	38,509(30.83)	8711(32.09)	13,491(39.36)
**Married or partnered**					
No	23,641(37.57)	5321(14.43)	32,501(26.02)	7512(27.67)	10,811(31.54)
Yes	39,291(62.43)	31,545(85.57)	92,425(73.98)	19,634(72.33)	23,462(68.46)
**Household wealth**					
Low	23,894(37.97)	12,108(32.84)	43,994(35.22)	8190(30.17)	13,290(38.78)
Middle	17,825(28.32)	12,711(34.48)	40,258(32.23)	9101(33.53)	9381(27.37)
High	21,213(33.71)	12,047(32.68)	40,674(32.56)	9855(36.30)	11,602(33.85)
**Smoking**					
No	54,062(85.91)	26,640(72.26)	104,254(83.45)	24,401(89.89)	30,151(87.97)
Yes	8870(14.09)	10,226(27.74)	20,672(16.55)	2745(10.11)	4122(12.03)
**Drinking**					
No	26,614(42.29)	20,406(55.35)	64,163(51.36)	3640(13.41)	25,708(75.01)
Yes	36,318(57.71)	16,460(44.65)	60,763(48.64)	23,506(86.59)	8565(24.99)
**Co-residence with children**					
No	58,389(92.78)	19,998(54.25)	101,421(81.18)	21,441(78.98)	10,102(29.48)
Yes	4543(7.22)	16,868(45.75)	23,505(18.82)	5705(21.02)	24,171(70.52)
**Internet use**					
No	26,423(41.99)	34,815(94.44)	59,699(47.79)	7506(27.65)	20,779(60.63)
Yes	36,509(58.01)	2051(5.56)	65,227(52.21)	19,640(72.35)	13,494(39.37)
Frailty index	18.62(10.86–30.06)	17.16(11.12–26.58)	13.97(8.28–23.62)	12.82(7.22–22.24)	16.18(9.23–27.13)
**Frailty status**					
Non-frailty	41,284(65.60)	26,407(71.63)	96,850(77.53)	21,522(79.28)	24,593(71.76)
Frailty	21,648(34.40)	10,459(28.37)	28,076(22.47)	5624(20.72)	9680(28.24)

*HRS* Health and Retirement Study; *CHARLS* China Health and Retirement Longitudinal Study; *SHARE* Survey of Health, Ageing and Retirement in Europe; *ELSA* English Longitudinal Study of Ageing; *MHAS* Mexican Health and Aging Study

According to Table 2, internet use was associated with a lower risk of frailty in all models. In fully adjusted model (Model 3), those association remained statistically significant in HRS (OR = 0.77, 95%CI: 0.74, 0.80), CHARLS (OR = 0.62, 95%CI: 0.54, 0.70), SHARE (OR = 0.47, 95%CI: 0.29, 0.76), ELSA (OR = 0.71, 95%CI: 0.66, 0.77), MHAS (OR = 0.81, 95%CI: 0.76, 0.85). After meta-analysis, the pooled result from five cohorts was significant (OR = 0.72, 95%CI: 0.67, 0.79). When the frailty index as an outcome variable was utilized in GEE models, participants with internet use had a lower frailty index, namely less healthy deficits (Table 3).

**Table 2:** The association of internet use and frailty status

	HRS		CHARLS		SHARE		ELSA		MHAS		Pooled	*P* value for heterogeneity
	OR(95%CI)	*P* value	OR(95%CI)	*P* value	OR(95%CI)	*P* value	OR(95%CI)	*P* value	OR(95%CI)	*P* value	OR(95%CI)	
Model 1	0.52(0.50,0.54)	< 0.001	0.44(0.39,0.49)	< 0.001	0.27(0.27,0.28)	< 0.001	0.43(0.40,0.46)	< 0.001	0.68(0.64,0.71)	< 0.001	0.45(0.31,0.65)	< 0.001
Model 2	0.59(0.57,0.62)	< 0.001	0.53(0.47,0.59)	< 0.001	0.43(0.42,0.45)	< 0.001	0.57(0.53,0.62)	< 0.001	0.72(0.68,0.76)	< 0.001	0.56(0.46,0.69)	< 0.001
Model 3	0.77(0.74,0.80)	< 0.001	0.62(0.54,0.70)	< 0.001	0.47(0.29,0.76)	0.002	0.71(0.66,0.77)	< 0.001	0.81(0.76,0.85)	< 0.001	0.72(0.67,0.79)	< 0.001

*HRS* Health and Retirement Study; *CHARLS* China Health and Retirement Longitudinal Study; *SHARE* Survey of Health, Ageing and Retirement in Europe; *ELSA* English Longitudinal Study of Ageing; *MHAS* Mexican Health and Aging Study; Model 1 was a crude model; In model 2, we accounted for age and gender; Model 3 was fully adjusted, further controlling educational levels, work for payment, married or partnered, household wealth, smoking, drinking, and co-residence with children

**Table 3:** The association of internet use and frailty index

	HRS		CHARLS		SHARE		ELSA		MHAS	
	β(95%CI)	*P* value	β(95%CI)	*P* value	β(95%CI)	*P* value	β(95%CI)	*P* value	β(95%CI)	*P* value
Model 1	-4.23(-4.46,-4.00)	< 0.001	-3.23(-3.62,-2.83)	< 0.001	-8.20(-8.40,-7.99)	< 0.001	-4.74(-5.15,-4.33)	< 0.001	-2.17(-2.46,-1.89)	< 0.001
Model 2	-2.98(-3.19,-2.76)	< 0.001	-2.13(-2.49,-1.77)	< 0.001	-4.65(-5.09,-4.22)	< 0.001	-2.82(-3.20,-2.45)	< 0.001	-1.54(-1.80,-1.28)	< 0.001
Model 3	-1.58(-1.79,-1.36)	< 0.001	-1.32(-1.69,-0.95)	< 0.001	-2.50(-2.79,-2.20)	< 0.001	-1.76(-2.13,-1.39)	< 0.001	-0.81(-1.08,-0.54)	< 0.001

*HRS* Health and Retirement Study; *CHARLS* China Health and Retirement Longitudinal Study; *SHARE* Survey of Health, Ageing and Retirement in Europe; *ELSA* English Longitudinal Study of Ageing; *MHAS* Mexican Health and Aging Study; Model 1 was a crude model; In model 2, we accounted for age and gender; Model 3 was fully adjusted, further controlling educational levels, work for payment, married or partnered, household wealth, smoking, drinking, and co-residence with children

In sensitivity analyses, the association in main results remained statistically significant among all cohort surveys after excluding participants with severe cognitive impairment and memory disease separately (Supplementary Tables 4 and Supplementary Table 5). In inverse probability weights GEE models, the associations were similar to the main results (Supplementary Table 6). Besides, we calculated the E-values, which represented the minimum strength that unmeasured confounding fully explained exposure-outcome association based on the risk ratio scale, to evaluate robustness in the association of internet use with frailty status (Supplementary Table 7). Again, internet use was inversely associated with poor physical health, function limitations, depression, and cognition score (Supplementary Table 8). Finally, after excluding participants with frailty at baseline, the association remained significant in HRS, CHARLS, SHARE, and MHAS, but not in ELSA (Supplementary Table 9).

**Fig. 2:**
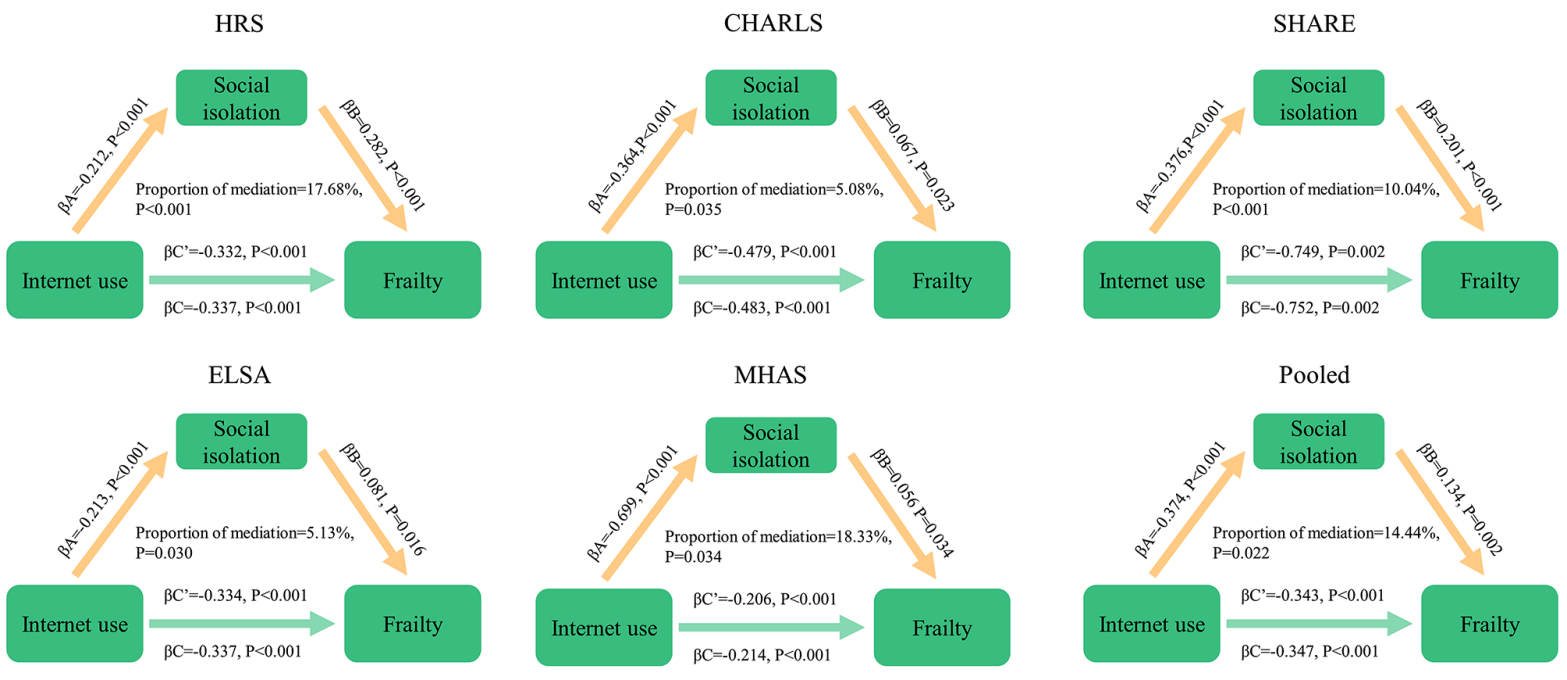
Association between internet use, frailty, and social isolation. βA represents the effects of internet use on social isolation; βB represents the effects of social isolation on frailty; βC’ represents the effects of internet use on frailty with social isolation; βC represents the effects of internet use on frailty without social isolation. HRS: Health and Retirement Study; CHARLS: China Health and Retirement Longitudinal Study; SHARE: Survey of Health, Ageing and Retirement in Europe; ELSA: English Longitudinal Study of Ageing; MHAS: Mexican Health and Aging Study

Mediation analysis pointed out that social isolation partially mediated the association between internet use and frailty status in HRS (17.68%, *p* < 0.001), CHARLS (5.08%, *p* = 0.035), SHARE (10.04%, *p* < 0.001). ELSA (5.13%, *p* = 0.030), and MHAS (18.33%, *p* = 0.034). After meta-analysis, social isolation still mediated the association of internet use with frailty status (Fig. 2).

**Fig. 3:**
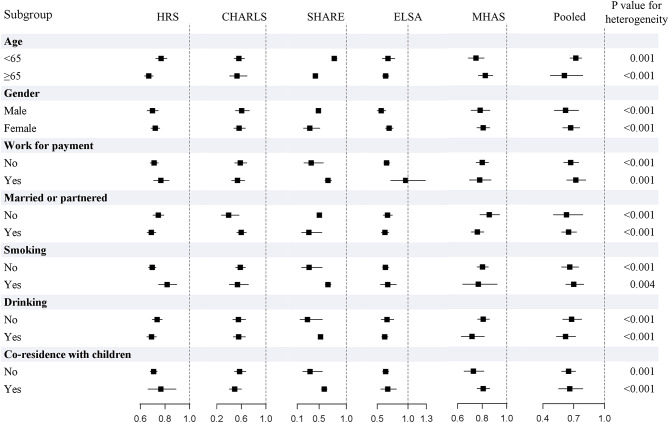
Stratified association of internet use and frailty status. *HRS* Health and Retirement Study; *CHARLS* China Health and Retirement Longitudinal Study; *SHARE* Survey of Health, Ageing and Retirement in Europe; *ELSA* English Longitudinal Study of Ageing; *MHAS* Mexican Health and Aging Study. All models adjusted age, gender, educational levels, work for payment, married or partnered, household wealth, smoking, drinking, and co-residence with children

Figure 3 and Supplementary Table 10 presented the potential difference in the effect of internet use across different subgroups. In general, the pooled results revealed a slightly stronger effect in participants aged 65 and over, male, not working for payment, not married or partnered, not smoking, drinking, and not co-residence with children.

## Discussion

In this study with harmonized cohorts, internet use rates ranged from 5.56% in China, 39.37% in Mexico to 58.01% in the United States, 72.35% in England, and 83.46% in Denmark (SAHRE). We found developed countries are more digitalized than developing countries. Besides, middle-aged and older participants with internet use reported a lower prevalence rate of frailty compared to those excluded from digital in both developed (HRS, SHARE, ELSA) and developing countries (CHARLS, MHAS).

To our knowledge, this study was the first to explore the longitudinal association between internet use and frailty status in middle-aged and old adults. Previous research has indicated that internet use was inversely associated with frailty in postmenopausal women [16]. However, the small sample size and cross-section design might limit the reliability and robustness of the findings. In addition, more studies focused on whether frailty status influenced internet use and reported inclusive conclusions, while it was not the concern of our study [34–36]. With the rapidly growing proportion of the population engaging in the internet over the past decade, researchers in the field of digital technology gradually explored the potential health effects of the internet [37]. Nevertheless, the rate of internet use in older adults remained relatively low, especially in developing countries such as China and Mexico. With technology advancing and population aging, the trend of the internet as a platform for providing health services and monitoring poor lifestyles was unavoidable worldwide. A scoping review supported digital health interventions among people would be beneficial for the frailty status, although the initial purposes of those interventions were various [38]. Population with frailty considered internet access maintained a social connection with friends and relatives and engagement in their daily lives, which reduced social isolation and loneliness [39]. Utilizing the available multi-country population-based cohorts, we could investigate whether the effect of internet use on frailty differed in the context of developed and developing countries as well as the mediating role of social isolation.

By including five longitudinal cohorts from 32 countries, internet use was discovered to be inversely associated with frailty in middle-aged and older adults. Previous studies found internet use had a protective effect on multiple chronic diseases (stroke, hypertension, eyesight, etc.), self-reported healthy status, functional limitations, depression, and cognition [9, 12–15, 21, 40–42]. However, few studies have combined those health deficits to evaluate the association between internet use and frailty. The present results indicated the protective effect was more pronounced in developed countries except for China. One possible explanation was that China experienced rapid economic development and increasing technological levels in 2011–2018. With the coverage of the internet, online health services such as purchasing medicines and asking for medical advice provided a chance to maintain self-health status in middle-aged and older adults [43]. Meanwhile, internet access as a common tool is quietly changing the traditional channels of obtaining medical services and work information. We also found the most notable association was reported in participants from SHARE and the highest rate of internet use in Denmark (SHARE). Compared to developing countries, more comprehensive health management systems and enriched online medical knowledge were implemented in developed countries. The findings suggested the requirements of enhancing internet access and refining online services to prevent the incidence of frailty, especially in developing countries. Moreover, bridging the digital divide in an aging society would reduce the health disparity in frailty status between developed and developing countries.

We further explored the potential mechanism, social isolation, to explain the association between internet use and frailty status at a population level. According to the mediation analysis in our study, the negative association of internet use with frailty was partially mediated by social isolation. The internet as a virtual world played a role in providing the available resources to bring people closer together. Active internet users effectively maintained connections with friends and expanded social networks, [44] which could reduce loneliness and depression, especially in older adults [45, 46]. At the same time, loneliness and social isolation would be confirmed to accelerate the progression of frailty based on the population from ELSA [47]. Previous studies reported that the internet facilitated interaction with relatives and friends, and improved engagement in social groups. Community-related organizations and volunteers [48, 49]. Social activities, especially high-frequency ones, significantly decreased the frailty among middle-aged and older adults from CHARLS [24]. In particular, The internet also provided opportunities for online social engagement and support in the special populations with disability or function limitations.

Besides, other possible mechanisms based on previous studies were able to interpret the current health effects of internet use on the prevention of frailty. Older adults expressed a positive mindset towards the internet infrastructure as an instrument of achieving convenience and relaxation [50]. Additionally, middle-aged and older adults also tended to search for healthy diets, exercise, online medical information, and remote health services because they were concerned about their health [18]. For instance, short health videos on social media platforms were extremely popular among Chinese aging women [51]. Handy and unrestricted internet access encouraged people to recognize healthcare knowledge regardless of time and place, which would be an inexpensive way to improve the life of quality, particularly among the population with health-related difficulties [52]. On the other hand, middle-aged and older adults expected to modify the unhealthy lifestyles associated with frailty through the internet [53]. Regular internet users were more inclined to adopt healthy lifestyles including vegetable and fruit eating, not smoking, and not binge drinking [54]. Notably, to prevent the incidence of frailty in this way would need a powerful self-management capability.

Although the present findings implicate that internet use was beneficial for preventing the incidence of frailty, what cannot be ignored is that the situation of problematic internet use and internet addiction might spread among older people. Previous studies also supported that excessive time spent on the internet had a high risk of depressive symptoms [55]. In addition, there was a large proportion population with lower educational attainment in developing countries, who contributed to less help from the internet. In particular, our study has shown that the low educational level and high rate of internet use existed simultaneously in Mexico, which explained the protective effect of internet use was minimal compared to other cohorts to some extent. Under-educated people might be excluded from healthy information websites because a high school level or greater reading ability was required [56]. In addition, based on the population from CHARLS, the low-literacy older adults were less likely to search for health services [57]. So, relevant strategies for internet education and health literacy promotion to encourage effective internet access should be underscored in developed and developing countries.

In summary, our findings have some public health implications and practical value. First, internet had the potential to provide various opportunities for preventing frailty in middle-aged and older adults. Relevant workers in health field should promote internet penetration and provide more easy-to-read health information, especially for under-educated older adults. Moreover, social isolation played a mediated role between internet use and frailty status, which implied that maintaining social connection and communication via the internet might be a crucial pathway to prevent frailty. Furthermore, Well-established evidence showed that digital interventions have successfully emerged as a vital part of healthcare service, which facilitated the utilization of medical resources. For instance, a systematic review of randomized controlled studies demonstrated that the behavioral intervention using mobile health applications to manage chronic diseases had promising aspects [58]. Another recent review revealed that e-health and m-health technologies increase physical activities, which could improve frailty status [59, 60]. Therefore, internet access among middle-aged and older adults would help build a healthy aging society.

Our study had some strengths. We included five national representative and longitudinal cohorts from developed and developing countries with large sample sizes. The five surveys were cross-cultural comparable at the beginning of the design. Besides, sufficient items from multiple repeated measurements to construct the frailty index and diverse sensitivity analyses enable the reliability and robustness of our results. Finally, The GEE models could effectively deal with the correlations from longitudinal panel data to reduce the error of the results.

Several limitations should be also aware in this study. First, recall biases were unavoidable because most variables selected for our study were determined based on self-reported information. Second, heterogeneity among HRS, CHARLS, SHARE, ELSA, and MHAS remained due to the different evaluations of internet use, social isolation, and frailty index, especially between MHAS and other cohorts. Specifically, internet use was measured at the household level in MHAS, but other cohorts were from the individual level. Meanwhile, the number and description of items constructing the index of social isolation and frailty were not the same. Third, the dimensions of internet use, including frequency and purpose, were not considered as the lack of data availability. Fourth, unmeasured confounding including sleep and genetic susceptibility remained while the E-value was calculated. Hence, further research should be implemented to confirm the benefit of internet use based on other populations and determine the bi-directional causation between internet use and frailty status. Additionally, the dynamic change in internet use and frailty status should be also explored, which contributed to healthy policy development.

## Conclusion

In conclusion, we found internet use inversely associated with frailty, as well as mediated by social isolation. With the increasing trend of internet use, the digital era has become inevitable even in middle-aged and older adults. Balancing the pros and cons of the internet should be carefully considered in developed and developing countries. Grasping the opportunity to avoid social isolation via the internet would decrease the incidence of frailty and address health concerns.

### Electronic supplementary material

Below is the link to the electronic supplementary material.

Supplementary Material 1

## Data Availability

The datasets generated and analyzed during the current study are available from the websites of HRS at https://hrs.isr.umich.edu/, CHARLS at https://charls.pku.edu.cn/en, SHARE at https://share-eric.eu/, ELSA at https://www.elsa-project.ac.uk/, and MHAS at http://www.mhasweb.org/.

## Abbreviations

CHARLS: China Health and Retirement Longitudinal Study
CIs: Confidence intervals
ELSA: English Longitudinal Study of Ageing
GEE: Generalized estimating equations
HRS: Health and Retirement Study
MHAS: Mexican Health and Aging Study
ORs: Odd ratios
SHARE: Survey of Health, Ageing and Retirement in Europe
