# Supplementary material for: Internet use and frailty in middle-aged and older adults: findings from developed and developing countries
Source: Global Health. 2024 Jul 3;20:53. doi: 10.1186/s12992-024-01056-6 (PMC11223306; doi:10.1186/s12992-024-01056-6)
Supplement: Supplementary file 1 — Supplementary Material 1 [file 12992_2024_1056_MOESM1_ESM.docx]

Content

[**Supplementary Figure 1** The flowcharts of participants selection in HRS, CHARLS, SHARE, ELSA, and MHAS. 2](#_Toc168252344)

[**Supplementary Table 1** The items used to construct the frailty index in HRS, CHARLS, SHARE, ELSA, and MHAS. 4](#_Toc168252345)

[**Supplementary Table 2** The items used to construct the index of social isolation in HRS, CHARLS, SHARE, ELSA, and MHAS. 6](#_Toc168252346)

[**Supplemental Figure 2** The distribution of frailty index in HRS, CHARLS, SHARE, ELSA, and MHAS. 7](#_Toc168252347)

[**Supplementary Table 3** The proportion of internet use and frailty across countries. 9](#_Toc168252348)

[**Supplementary Table 4** The association of internet use and frailty after excluding participants with severe cognitive impairment at baseline. 10](#_Toc168252349)

[**Supplementary Table 5** The association of internet use and frailty after excluding participants with memory disease at baseline. 11](#_Toc168252350)

[**Supplementary Table 6** The association of internet use and frailty using the inverse probability weights. 12](#_Toc168252351)

[**Supplementary Table 7** The E-value of association of internet use and frailty. 13](#_Toc168252352)

[**Supplementary Table** **8** The association of internet use and specific domains of frailty. 14](#_Toc168252353)

[**Supplementary Table** **9** The association of internet use and frailty using COX regression. 15](#_Toc168252354)

[**Supplementary Table** **10** Stratified association of internet use and frailty status. 16](#_Toc168252355)

**Supplementary Figure 1** The flowcharts of participants selection in HRS, CHARLS, SHARE, ELSA, and MHAS.

**
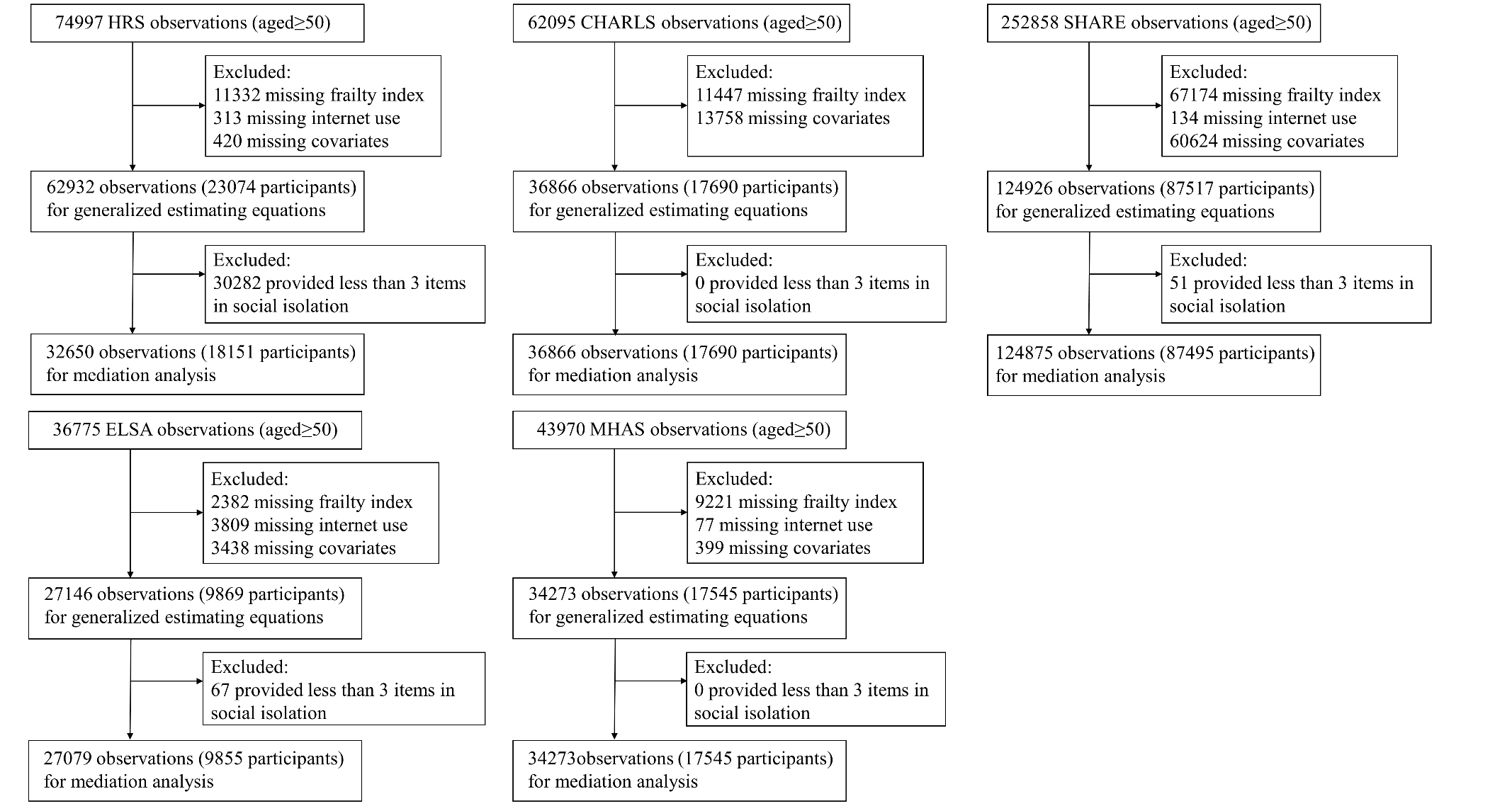
**

HRS: Health and Retirement Study; CHARLS: China Health and Retirement Longitudinal Study; SHARE: Survey of Health, Ageing and Retirement in Europe; ELSA: English Longitudinal Study of Ageing; MHAS: Mexican Health and Aging Study.

**Supplementary Table 1** The items used to construct the frailty index in HRS, CHARLS, SHARE, ELSA, and MHAS.

| No | Description of the items | | | | | Cut-off value |
| --- | --- | --- | --- | --- | --- | --- |
|  | HRS (2012-2018) | CHARLS (2011-2018) | SHARE (2013-2019) | ELSA (2012-2018) | MHAS (2012-2018) |  |
| 1 | Self-reported diagnosed hypertension by doctors | | | | | Yes=1, No=0 |
| 2 | Self-reported diagnosed diabetes by doctors | | | | | Yes=1, No=0 |
| 3 | Self-reported diagnosed heart disease by doctors | | | | Self-reported diagnosed heart attack by doctors | Yes=1, No=0 |
| 4 | Self-reported diagnosed stroke by doctors | | | | | Yes=1, No=0 |
| 5 | Self-reported diagnosed cancer by doctors | | | | | Yes=1, No=0 |
| 6 | Self-reported diagnosed arthritis by doctors | | | | | Yes=1, No=0 |
| 7 | Self-reported diagnosed chronic lung disease by doctors | | | | Self-reported diagnosed respiratory disease by doctors | Yes=1, No=0 |
| 8 | Self-reported diagnosed any emotional, nervous, or psychiatric problems by doctors | | | | Unavailable | Yes=1, No=0 |
| 9 | Self-reported diagnosed memory-related disease, including Alzheimer’s disease or dementia, organic brain senility, or other serious memory impairment | | | | Unavailable | Yes=1, No=0 |
| 10 | Self-reported general eyesight | Self-reported eyesight for seeing thing up close | | Self-reported general eyesight | | Poor or blind=1, fair=0.75, good=0.5, very good=0.25, excellent=0 |
| 11 | Self-reported hearing while wearing hearing aid or as usual | | | | | Poor or deaf=1, fair=0.75, good=0.5, very good=0.25, excellent=0 |
| 12 | Self-reported healthy status | | | | | Poor=1, fair=0.75, good=0.5, very good=0.25, excellent=0 |
| 13 | BADL: Any difficulty in dressing | | | | | Yes=1, No=0 |
| 14 | BADL: Any difficulty in bathing or showering | | | | | Yes=1, No=0 |
| 15 | BADL: Any difficulty in eating | | | | | Yes=1, No=0 |
| 16 | BADL: Any difficulty in getting in/out bed | | | | | Yes=1, No=0 |
| 17 | BADL: Any difficulty in using the toilet | | | | | Yes=1, No=0 |
| 18 | IADL: Any difficulty in managing money | | | | | Yes=1, No=0 |
| 19 | IADL: Any difficulty in taking medications | | | | | Yes=1, No=0 |
| 20 | IADL: Any difficulty in shopping | | | | | Yes=1, No=0 |
| 21 | IADL: Any difficulty in preparing meals | | | | | Yes=1, No=0 |
| 22 | Mobility: Any difficulty in walking 1 block | | | | | Yes=1, No=0 |
| 23 | Mobility: Any difficulty in getting up from a chair after sitting for long periods | | | | | Yes=1, No=0 |
| 24 | Mobility: Any difficulty in climbing several flights of stairs without resting | | | | | Yes=1, No=0 |
| 25 | Mobility: Any difficulty in lifting or carrying weights over 10 pounds | | | | | Yes=1, No=0 |
| 26 | Mobility: Any difficulty in picking up a coin from table | | | | | Yes=1, No=0 |
| 27 | Mobility: Any difficulty in stooping kneeling or crouching | | | | | Yes=1, No=0 |
| 28 | Mobility: Any difficulty in reaching arms above shoulder level | | | | | Yes=1, No=0 |
| 29 | Depression: CESD-8 (ranging from 0 to 8) | Depression: CESD-10 (ranging from 0 to 30) | Depression: EURO (ranging from 0 to 12) | Depression: CESD-8 (ranging from 0 to 8) | Depression: CESD-9 (ranging from 0 to 9) | CESD-8≥3, CESD-9≥5, CESD-10≥10, EURO≥4=1, CESD-8<3, CESD-9<5, CESD-10<10, EURO<4=0 |
| 30 | Cognition: (total score-(immediate and delayed word recall + date naming + serial 7’s))/total score | | | | | Continuous, from 0 to 1 |

HRS, Health and Retirement Study; CHARLS, China Health and Retirement Longitudinal Study; SHARE, Survey of Health, Ageing and Retirement in Europe; ELSA, English Longitudinal Study of Ageing; MHAS, Mexican Health and Aging Study; CESD, Center for Epidemiologic Studies Depression Scale.

Cognition test consists of four components, including immediate and delayed word recall, date naming and serial 7’s. For word recall, participants are required to recite 10 words in HRS, CHARLS, SHARE, and ELSA, and 8 words in MHAS. For date naming, participants are asked whether they could remember the date of that day (day of week, day of month, month, and year in HRS, CHARLS, SHARE, and ELSA, and day of month, month, and year in MHAS). For serial 7’s, participants are required to make five calculations and answer how much is 100 minus 7. However, serial 7’s test could be unavailable in SHARE 2013 and MHAS 2012, so we only did not include the component in the two waves. One point is given for each right answer, and the cognition score is calculated by the formula: (total score-(immediate and delayed word recall + date naming + serial 7’s))/total score. Total score refers to the theoretical maximum score of all tests. Hence, a higher cognition score indicated lower cognition function.

**Supplementary Table 2** The items used to construct the index of social isolation in HRS, CHARLS, SHARE, ELSA, and MHAS.

| Item | Description of the items | | | | | Cut-off value |
| --- | --- | --- | --- | --- | --- | --- |
|  | HRS | CHARLS | SHARE | ELSA | MHAS |  |
| Unmarried | Would you say you are currently married, separated, divorced, widowed, or never married? | | | | | Unmarried=1, other marital status=0 |
| Live alone | The number of residents in the household including the respondent. If only one resident indicates the respondent lives alone. More than one resident indicates the respondent lives with others.  The variable is not directly asked in questionnaire, but provides the relevant value in household roster or derived file. | | | | | Live alone=1, Live with others=0 |
| Contact with children | Whether the respondent was weekly contact in person, phone or email with at least one child in past year? | | | | | No=1, Yes=0 |
| Contact with parents, relatives or friends | Whether the respondent was weekly contact in person, phone or email with parents, relatives or friends | Whether the respondent was weekly contact in person, phone or email with parents | | Whether the respondent was weekly contact in person, phone or email with relatives or friends | | No=1, Yes=0 |
| Ever participate in any groups, clubs, or other organizations | (i) Go to a sport, social, or other club; (ii) Attend meetings of non-religious  organizations, such as political,  community, or other interest groups? | (i) Played Ma-jong, played chess, played cards, or went to community club; (ii) Went to a sport, social, or other kind of club (iii) Took part in a community-related organization | (i) Gone to a sport, social or other kind of club; (ii) Taken part in a political or community-related organization | (i) Political or community-related organization (ii) Tenants groups, resident groups; (iii) Education, arts or music groups or evening classes; | (i) Work as a volunteer or help a non-profit organization  without pay or compensation; (ii)  Attend a sporting or social club | None=1, At least one=0 |

HRS, Health and Retirement Study; CHARLS, China Health and Retirement Longitudinal Study; SHARE, Survey of Health, Ageing and Retirement in Europe; ELSA, English Longitudinal Study of Ageing; MHAS, Mexican Health and Aging Study.

**Supplemental Figure 2** The distribution of frailty index in HRS, CHARLS, SHARE, ELSA, and MHAS.


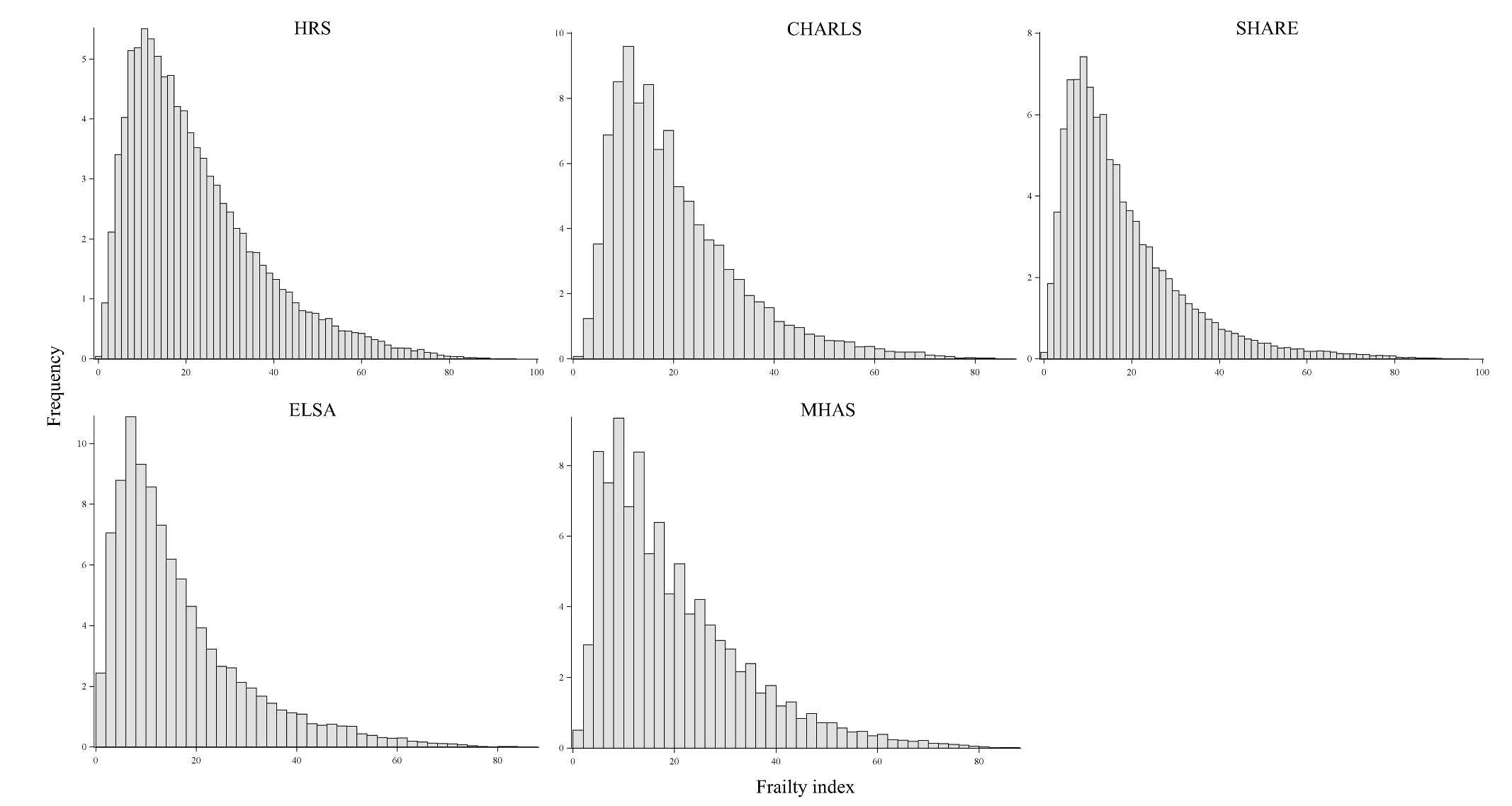


HRS, Health and Retirement Study; CHARLS, China Health and Retirement Longitudinal Study; SHARE, Survey of Health, Ageing and Retirement in Europe; ELSA, English Longitudinal Study of Ageing; MHAS, Mexican Health and Aging Study.

**Supplementary Table 3** The proportion of internet use and frailty across countries.

|  |  | Internet use | Frailty |
| --- | --- | --- | --- |
| HRS | United States | 36509(58.01%) | 21648(34.40%) |
| CHARLS | China | 2051(5.56%) | 10459(28.37%) |
| SHARE | Austria | 2882(50.62%) | 1227(21.55%) |
|  | German | 5155(59.53%) | 1768(20.42%) |
|  | Sweden | 5534(76.27%) | 1007(13.88%) |
|  | Netherlands | 4623(79.46%) | 751(12.91%) |
|  | Spain | 2769(31.81%) | 2548(29.27%) |
|  | Italy | 2842(32.7%) | 2187(25.17%) |
|  | France | 4464(58.85%) | 1926(25.39%) |
|  | Denmark | 5781(83.46%) | 871(12.57%) |
|  | Greece | 2091(31.05%) | 1373(20.39%) |
|  | Switzerland | 3667(70.24%) | 584(11.19%) |
|  | Belgium | 5773(64.48%) | 1906(21.29%) |
|  | Israel | 1534(51.34%) | 766(25.64%) |
|  | Czech Republic | 3990(46.78%) | 2159(25.31%) |
|  | Poland | 1041(33.45%) | 1086(34.90%) |
|  | Luxembourg | 1702(64.20%) | 480(18.11%) |
|  | Hungary | 326(42.78%) | 260(34.12%) |
|  | Portugal | 42(31.11%) | 31(22.96%) |
|  | Slovenia | 2640(39.84%) | 1555(23.47%) |
|  | Estonia | 3998(46.09%) | 2932(33.80%) |
|  | Croatia | 1133(32.16%) | 839(23.81%) |
|  | Lithuania | 638(45.34%) | 401(28.5%) |
|  | Bulgaria | 192(22.15%) | 237(27.34%) |
|  | Cyprus | 111(21.51%) | 140(27.13%) |
|  | Finland | 874(76.80%) | 188(16.52%) |
|  | Latvia | 365(47.9%) | 209(27.43%) |
|  | Malta | 341(43.89%) | 84(10.81%) |
|  | Romania | 305(24.42%) | 416(33.31%) |
|  | Slovakia | 414(42.81%) | 145(14.99%) |
| ELSA | England | 19640(72.35%) | 5624(20.72%) |
| MHAS | Mexico | 13494(39.37%) | 9680(28.24%) |

HRS, Health and Retirement Study; CHARLS, China Health and Retirement Longitudinal Study; SHARE, Survey of Health, Ageing and Retirement in Europe; ELSA, English Longitudinal Study of Ageing; MHAS, Mexican Health and Aging Study.

**Supplementary Table 4** The association of internet use and frailty after excluding participants with severe cognitive impairment at baseline.

|  | HRS | | CHARLS | | SHARE | | ELSA | | MHAS | |
| --- | --- | --- | --- | --- | --- | --- | --- | --- | --- | --- |
|  | OR(95%CI) | P value | OR(95%CI) | P value | OR(95%CI) | P value | OR(95%CI) | P value | OR(95%CI) | P value |
| Model 1 | 0.55(0.53,0.58) | <0.001 | 0.60(0.51,0.71) | <0.001 | 0.28(0.27,0.29) | <0.001 | 0.46(0.43,0.50) | <0.001 | 0.73(0.69,0.77) | <0.001 |
| Model 2 | 0.62(0.60,0.65) | <0.001 | 0.59(0.49,0.72) | <0.001 | 0.42(0.41,0.44) | <0.001 | 0.60(0.55,0.65) | <0.001 | 0.74(0.70,0.79) | <0.001 |
| Model 3 | 0.78(0.74,0.82) | <0.001 | 0.70(0.57,0.86) | 0.001 | 0.31(0.12,0.78) | 0.013 | 0.73(0.67,0.79) | <0.001 | 0.82(0.77,0.88) | <0.001 |

HRS, Health and Retirement Study; CHARLS, China Health and Retirement Longitudinal Study; SHARE, Survey of Health, Ageing and Retirement in Europe; ELSA, English Longitudinal Study of Ageing; MHAS, Mexican Health and Aging Study; Compared to participants with the same level of education, those who have 1.5 standard deviations below the mean of cognitive score in three or four tests are classified as severe cognitive impairment; Model 1 was a crude model; In model 2, we accounted for age and gender; Model 3 was fully adjusted, further controlling educational levels, work for payment, married or partnered, household wealth, smoking, drinking, and co-residence with children.

**Supplementary Table 5** The association of internet use and frailty after excluding participants with memory disease at baseline.

|  | HRS | | CHARLS | | SHARE | | ELSA | | MHAS | |
| --- | --- | --- | --- | --- | --- | --- | --- | --- | --- | --- |
|  | OR(95%CI) | P value | OR(95%CI) | P value | OR(95%CI) | P value | OR(95%CI) | P value | OR(95%CI) | P value |
| Model 1 | 0.56(0.53,0.58) | <0.001 | 0.63(0.53,0.74) | <0.001 | 0.28(0.27,0.30) | <0.001 | 0.46(0.43,0.50) | <0.001 | 0.68(0.64,0.71) | <0.001 |
| Model 2 | 0.62(0.60,0.65) | <0.001 | 0.62(0.51,0.74) | <0.001 | 0.43(0.42,0.45) | <0.001 | 0.60(0.55,0.65) | <0.001 | 0.72(0.68,0.76) | <0.001 |
| Model 3 | 0.78(0.74,0.81) | <0.001 | 0.72(0.59,0.88) | 0.002 | 0.31(0.12,0.80) | 0.015 | 0.73(0.67,0.79) | <0.001 | 0.81(0.76,0.85) | <0.001 |

HRS, Health and Retirement Study; CHARLS, China Health and Retirement Longitudinal Study; SHARE, Survey of Health, Ageing and Retirement in Europe; ELSA, English Longitudinal Study of Ageing; MHAS, Mexican Health and Aging Study; Model 1 was a crude model; In model 2, we accounted for age and gender; Model 3 was fully adjusted, further controlling educational levels, work for payment, married or partnered, household wealth, smoking, drinking, and co-residence with children.

**Supplementary Table 6** The association of internet use and frailty using the inverse probability weights.

|  | HRS | | CHARLS | | SHARE | | ELSA | | MHAS | |
| --- | --- | --- | --- | --- | --- | --- | --- | --- | --- | --- |
|  | OR(95%CI) | P value | OR(95%CI) | P value | OR(95%CI) | P value | OR(95%CI) | P value | OR(95%CI) | P value |
| Model 3 | 0.76(0.72,0.80) | <0.001 | 0.52(0.42,0.65) | <0.001 | 0.56(0.51,0.61) | <0.001 | 0.66(0.60,0.72) | <0.001 | 0.79(0.75,0.84) | <0.001 |

HRS, Health and Retirement Study; CHARLS, China Health and Retirement Longitudinal Study; SHARE, Survey of Health, Ageing and Retirement in Europe; ELSA, English Longitudinal Study of Ageing; MHAS, Mexican Health and Aging Study; Weights were calculated based on missing at random assumption using age, gender, educational levels, work for payment, married or partnered, smoking, drinking, household wealth, and co-residence with children; Models were adjusted for some covariates including age, gender, educational levels, work for payment, married or partnered, smoking, drinking, household wealth, and co-residence with children

**Supplementary Table 7** The E-value of association of internet use and frailty.

|  | HRS | | CHARLS | | SHARE | | ELSA | | MHAS | |
| --- | --- | --- | --- | --- | --- | --- | --- | --- | --- | --- |
|  | E-value | CI limit | E-value | CI limit | E-value | CI limit | E-value | CI limit | E-value | CI limit |
| Model 1 | 2.119 | 2.062 | 2.382 | 2.211 | 3.258 | 3.187 | 2.420 | 2.311 | 1.617 | 1.538 |
| Model 2 | 1.929 | 1.856 | 2.090 | 1.929 | 2.420 | 2.346 | 1.980 | 1.856 | 1.597 | 1.500 |
| Model 3 | 1.538 | 1.481 | 1.856 | 1.678 | 2.277 | 1.558 | 1.658 | 1.538 | 1.444 | 1.331 |

HRS, Health and Retirement Study; CHARLS, China Health and Retirement Longitudinal Study; SHARE, Survey of Health, Ageing and Retirement in Europe; ELSA, English Longitudinal Study of Ageing; MHAS, Mexican Health and Aging Study; Model 1 was a crude model; In model 2, we accounted for age and gender; Model 3 was fully adjusted, further controlling educational levels, work for payment, married or partnered, household wealth, smoking, drinking, and co-residence with children.

**Supplementary Table** **8** The association of internet use and specific domains of frailty.

|  | HRS | | CHARLS | | SHARE | | ELSA | | MHAS | |
| --- | --- | --- | --- | --- | --- | --- | --- | --- | --- | --- |
| Domains | OR/β(95%CI) | P value | OR/β(95%CI) | P value | OR/β(95%CI) | P value | OR/β(95%CI) | P value | OR/β(95%CI) | P value |
| Poor physical health | 0.86(0.83,0.89) | <0.001 | 0.88(0.81,0.97) | 0.007 | 0.65(0.48,0.89) | 0.007 | 0.91(0.85,0.98) | 0.009 | 0.99(0.95,1.03) | 0.624 |
| Function limitations | 0.69(0.66,0.72) | <0.001 | 0.45(0.38,0.53) | <0.001 | 0.61(0.59,0.64) | <0.001 | 0.69(0.64,0.76) | <0.001 | 0.81(0.77,0.86) | <0.001 |
| Depression | 0.75(0.72,0.79) | <0.001 | 0.69(0.62,0.77) | <0.001 | 0.73(0.71,0.75) | <0.001 | 0.72(0.66,0.79) | <0.001 | 0.66(0.62,0.69) | <0.001 |
| Cognition score (continue) | -0.048(-0.050,-0.045) | <0.001 | -0.069(-0.076,-0.062) | <0.001 | -0.081(-0.097,-0.066) | <0.001 | -0.057(-0.062,-0.052) | <0.001 | -0.041(-0.044,-0.037) | <0.001 |

HRS, Health and Retirement Study; CHARLS, China Health and Retirement Longitudinal Study; SHARE, Survey of Health, Ageing and Retirement in Europe; ELSA, English Longitudinal Study of Ageing; MHAS, Mexican Health and Aging Study;

The physical health conditions included hypertension, diabetes, heart diseases, stroke, cancer, arthritis, chronic lung disease, psychiatric problems, memory-related disease, eyesight, hearing and self-reported health status; Function limitations included all the items of BADL, IADL and mobility. For physical health conditions and function limitations, we calculated the score by sum of present deficits divided by the sum of items and then multiplied by 100. Hence, Participants with poor physical health or function limitations were defined as the score ≥ 25;

Cognition test consists of four components, including immediate and delayed word recall, date naming and serial 7’s. For word recall, participants are required to recite 10 words in HRS, CHARLS, SHARE, and ELSA, and 8 words in MHAS. For date naming, participants are asked whether they could remember the date of that day (day of week, day of month, month, and year in HRS, CHARLS, SHARE, and ELSA, and day of month, month, and year in MHAS). For serial 7’s, participants are required to make five calculations and answer how much is 100 minus 7. However, serial 7’s test could be unavailable in SHARE 2013 and MHAS 2012, so we only did not include the component in the two waves. One point is given for each right answer, and the cognition score is calculated by the formula: (total score-(immediate and delayed word recall + date naming + serial 7’s))/total score. Total score refers to the theoretical maximum score of all tests. Hence, a higher cognition score indicated lower cognition function.

Models were adjusted for some covariates including age, gender, educational levels, work for payment, married or partnered, smoking, drinking, household wealth, and co-residence with children.

**Supplementary Table** **9** The association of internet use and frailty using COX regression.

|  | HRS | | CHARLS | | SHARE | | ELSA | | MHAS | |
| --- | --- | --- | --- | --- | --- | --- | --- | --- | --- | --- |
|  | HR(95%CI) | P value | HR(95%CI) | P value | HR(95%CI) | P value | HR(95%CI) | P value | HR(95%CI) | P value |
| Model 1 | 0.48(0.45,0.52) | <0.001 | 0.15(0.09,0.26) | <0.001 | 0.35(0.33,0.36) | <0.001 | 0.44(0.39,0.50) | <0.001 | 0.60(0.54,0.66) | <0.001 |
| Model 2 | 0.56 (0.52,0.61) | <0.001 | 0.19(0.11,0.32) | <0.001 | 0.51(0.49,0.54) | <0.001 | 0.69(0.60,0.79) | <0.001 | 0.68(0.61,0.75) | <0.001 |
| Model 3 | 0.81(0.74,0.88) | <0.001 | 0.25(0.15,0.44) | <0.001 | 0.71(0.67,0.75) | <0.001 | 0.90(0.78,1.05) | 0.168 | 0.78(0.70,0.87) | <0.001 |

HRS, Health and Retirement Study; CHARLS, China Health and Retirement Longitudinal Study; SHARE, Survey of Health, Ageing and Retirement in Europe; ELSA, English Longitudinal Study of Ageing; MHAS, Mexican Health and Aging Study; HR, Hazard Risk; Model 1 was a crude model; In model 2, we accounted for age and gender; Model 3 was fully adjusted, further controlling educational levels, work for payment, married or partnered, household wealth, smoking, drinking, and co-residence with children.

**Supplementary Table** **10** Stratified association of internet use and frailty status.

|  | HRS | CHARLS | SHARE | ELSA | MHAS | Pooled | P value for heterogeneity |
| --- | --- | --- | --- | --- | --- | --- | --- |
| Age |  |  |  |  |  |  |  |
| P for interaction | 0.146 | 0.500 | 0.005 | 0.434 | 0.254 |  |  |
| <65 | 0.77(0.72,0.82) | 0.56(0.48,0.65) | 0.78(0.73,0.83) | 0.67(0.58,0.79) | 0.75(0.69,0.82) | 0.72(0.66,0.78) | 0.001 |
| ≥65 | 0.67(0.63,0.71) | 0.53(0.41,0.69) | 0.43(0.41,0.46) | 0.63(0.58,0.69) | 0.83(0.77,0.89) | 0.61(0.47,0.79) | <0.001 |
| Gender |  |  |  |  |  |  |  |
| P for interaction | 0.548 | 0.541 | 0.188 | 0.017 | 0.971 |  |  |
| Male | 0.70(0.65,0.75) | 0.60(0.50,0.73) | 0.49(0.46,0.52) | 0.56(0.50,0.64) | 0.78(0.71,0.87) | 0.62(0.51,0.75) | <0.001 |
| Female | 0.72(0.68,0.76) | 0.56(0.47,0.67) | 0.33(0.21,0.52) | 0.69(0.63,0.76) | 0.81(0.75,0.86) | 0.67(0.59,0.76) | <0.001 |
| Work for payment |  |  |  |  |  |  |  |
| P for interaction | 0.007 | 0.625 | 0.006 | 0.001 | 0.330 |  |  |
| No | 0.71(0.68,0.75) | 0.58(0.49,0.69) | 0.36(0.22,0.58) | 0.65(0.60,0.71) | 0.80(0.75,0.85) | 0.67(0.60,0.75) | <0.001 |
| Yes | 0.77(0.71,0.83) | 0.54(0.44,0.66) | 0.66(0.60,0.73) | 0.96(0.71,1.29) | 0.78(0.69,0.87) | 0.72(0.63,0.82) | 0.001 |
| Married or partnered |  |  |  |  |  |  |  |
| P for interaction | 0.062 | 0.169 | 0.314 | 0.259 | 0.034 |  |  |
| No | 0.74(0.70,0.79) | 0.39(0.27,0.57) | 0.50(0.47,0.54) | 0.67(0.59,0.75) | 0.86(0.78,0.94) | 0.63(0.50,0.79) | <0.001 |
| Yes | 0.69(0.65,0.73) | 0.60(0.52,0.69) | 0.31(0.18,0.56) | 0.62(0.56,0.69) | 0.76(0.71,0.82) | 0.65(0.58,0.73) | <0.001 |
| Smoking |  |  |  |  |  |  |  |
| P for interaction | 0.001 | 0.490 | 0.002 | 0.971 | 0.824 |  |  |
| No | 0.70(0.66,0.73) | 0.58(0.50,0.67) | 0.31(0.18,0.56) | 0.63(0.58,0.69) | 0.80(0.76,0.85) | 0.66(0.58,0.75) | <0.001 |
| Yes | 0.82(0.74,0.90) | 0.54(0.40,0.72) | 0.66(0.61,0.72) | 0.67(0.55,0.81) | 0.77(0.64,0.92) | 0.70(0.62,0.80) | 0.004 |
| Drinking |  |  |  |  |  |  |  |
| P for interaction | 0.037 | 0.922 | 0.030 | 0.177 | 0.912 |  |  |
| No | 0.74(0.69,0.78) | 0.55(0.46,0.67) | 0.29(0.14,0.56) | 0.66(0.56,0.77) | 0.81(0.76,0.86) | 0.68(0.59,0.78) | <0.001 |
| Yes | 0.69(0.65,0.73) | 0.56(0.46,0.67) | 0.52(0.50,0.55) | 0.62(0.57,0.68) | 0.72(0.63,0.82) | 0.62(0.53,0.72) | <0.001 |
| Co-residence with children |  |  |  |  |  |  |  |
| P for interaction | 0.053 | 0.275 | 0.371 | 0.596 | 0.218 |  |  |
| No | 0.71(0.68,0.74) | 0.57(0.48,0.68) | 0.33(0.20,0.56) | 0.63(0.58,0.69) | 0.73(0.65,0.82) | 0.65(0.58,0.72) | 0.001 |
| Yes | 0.77(0.66,0.89) | 0.49(0.40,0.61) | 0.59(0.54,0.65) | 0.67(0.55,0.81) | 0.81(0.76,0.86) | 0.66(0.55,0.79) | <0.001 |

HRS, Health and Retirement Study; CHARLS, China Health and Retirement Longitudinal Study; SHARE, Survey of Health, Ageing and Retirement in Europe; ELSA, English Longitudinal Study of Ageing; MHAS, Mexican Health and Aging Study; Models were adjusted for some covariates including age, gender, educational levels, work for payment, married or partnered, smoking, drinking, household wealth, and co-residence with children.
